# Supplementary material for: Polyketide synthase gene clusters in the ericoid mycorrhizal fungus Oidiodendron maius: genome-wide prediction, expression profiling, and functional characterization of knockout mutants
Source: Front Plant Sci. 2026 Sep 16;17:1924430. doi: 10.3389/fpls.2026.1924430 (PMC13623903; doi:10.3389/fpls.2026.1924430)
Supplement: Supplementary file 1 [file SupplementaryFile1.zip › Supplementary Materials/Supplementary Folder 1.zip!/Oidma1_AssemblyScaffolds_Repeatmasked/knownclusterblast/cluster14/jgi.p_Oidma1_102289_mibig_hits.html]

| MIBiG Protein | Description | MIBiG Cluster | MiBiG Product | % ID | % Coverage | BLAST Score | E-value |
| --- | --- | --- | --- | --- | --- | --- | --- |
| ACI30652.1 | cyclophilin-type\_peptidyl-prolyl\_cis-trans\_isomerase | BGC0000313 | Beauvericin\_biosynthetic\_gene\_cluster | 72 | 99.8381877023 | 932 | 2.2e-271 |
| CAH94365.1 | putative\_peptidyl-prolyl\_cis-trans\_isomerase\_A | BGC0000717 | Streptomycin\_biosynthetic\_gene\_cluster | 54 | 20.71197411 | 135 | 2.3e-31 |
